# Obesity and Life Expectancy with and without Diabetes in Adults Aged 55 Years and Older in the Netherlands: A Prospective Cohort Study

**DOI:** 10.1371/journal.pmed.1002086

**Published:** 2016-07-19

**Authors:** Klodian Dhana, Jana Nano, Symen Ligthart, Anna Peeters, Albert Hofman, Wilma Nusselder, Abbas Dehghan, Oscar H. Franco

**Affiliations:** 1 Department of Epidemiology, Erasmus MC, University Medical Center Rotterdam, Rotterdam, the Netherlands; 2 Deakin University, Geelong, Victoria, Australia; 3 Department of Epidemiology, Harvard T. H. Chan School of Public Health, Boston, Massachusetts, United States of America; 4 Public Health Erasmus MC, University Medical Center Rotterdam, Rotterdam, the Netherlands; University of Cambridge, UNITED KINGDOM

## Abstract

**Background:**

Overweight and obesity are associated with increased risk of type 2 diabetes. Limited evidence exists regarding the effect of excess weight on years lived with and without diabetes. We aimed to determine the association of overweight and obesity with the number of years lived with and without diabetes in a middle-aged and elderly population.

**Methods and Findings:**

The study included 6,499 individuals (3,656 women) aged 55 y and older from the population-based Rotterdam Study. We developed a multistate life table to calculate life expectancy for individuals who were normal weight, overweight, and obese and the difference in years lived with and without diabetes. For life table calculations, we used prevalence, incidence rate, and hazard ratios (HRs) for three transitions (healthy to diabetes, healthy to death, and diabetes to death), stratifying by body mass index (BMI) at baseline and adjusting for confounders. During a median follow-up of 11.1 y, we observed 697 incident diabetes events and 2,192 overall deaths. Obesity was associated with an increased risk of developing diabetes (HR: 2.13 [*p* < 0.001] for men and 3.54 [*p* < 0.001] for women). Overweight and obesity were not associated with mortality in men and women with or without diabetes. Total life expectancy remained unaffected by overweight and obesity. Nevertheless, men with obesity aged 55 y and older lived 2.8 (95% CI −6.1 to −0.1) fewer y without diabetes than normal weight individuals, whereas, for women, the difference between obese and normal weight counterparts was 4.7 (95% CI −9.0 to −0.6) y. Men and women with obesity lived 2.8 (95% CI 0.6 to 6.2) and 5.3 (95% CI 1.6 to 9.3) y longer with diabetes, respectively, compared to their normal weight counterparts. Since the implications of these findings could be limited to middle-aged and older white European populations, our results need confirmation in other populations.

**Conclusions:**

Obesity in the middle aged and elderly is associated with a reduction in the number of years lived free of diabetes and an increase in the number of years lived with diabetes. Those extra years lived with morbidity might place a high toll on individuals and health care systems.

## Abstract 2

Klodian Dhana and colleagues investigate the life expectancy for individuals who are normal weight, overweight, and obese and the difference in years lived with and without diabetes.

## Author Summary

**Why Was This Study Done?:**

- Obesity, which has contributed to a dramatic increase in type 2 diabetes, is one of today’s highest public health concerns. Measures of lifetime consequences such as life expectancy with and without diabetes are necessary to inform clinical care, patients, and health care policy to facilitate the understanding of the consequences of being overweight or obese.

**What Did the Researchers Do and Find?:**

- Using data from a well-defined European population with long-term follow-up, we calculated life expectancy for individuals who were normal weight, overweight, and obese and the difference in years lived with and without diabetes.
- We constructed three different health states: free of diabetes, diabetes, and death. We further evaluated how participants in our study moved from one state to another (incident diabetes, incident mortality among diabetics, and mortality among nondiabetics) to assess the difference in risk of mortality and diabetes among individuals 55 y and older by different categories of body mass index (BMI).
- There were no differences in total life expectancy by body weight status. Nevertheless, men with obesity lived 2.8 y fewer free of diabetes than their normal weight counterparts, whereas, for women, the difference between obese and normal weight subjects was 4.7 y. Moreover, men and women with obesity lived 2.8 and 5.3 more y with diabetes compared to normal weight counterparts.

**What Do These Findings Mean?:**

- Obesity increases the risk of developing diabetes earlier in life and the amount of years individuals live with diabetes. As long as the obesity epidemic continues, we will observe more individuals living with diabetes and for a longer period of time. Our findings underscore the importance of preventing and treating obesity for clinicians, patients, and policy makers.

## Introduction

Overweight and obesity, which have contributed to a dramatic increase in type 2 diabetes, are one of today’s highest public health concerns [1,2]. Previous estimates of the effect of obesity in diabetes have been limited to absolute risks or lifetime risk without combining information about quantity and quality of remaining years lived with or without the diabetes, raising a gap in the intuitive understanding of risk and impact communicated among doctors and patients [3]. Complementing current knowledge with comparative measures of the long-term dimensions of disease, such as life expectancy, provides information on different scenarios, including whether, for example, years with disease are increasing but the proportion of life spent free of disease is increasing or decreasing. Moreover, quantification of these estimates has been extensively recommended to help inform public health interventions [4].

Studies evaluating the association between obesity and life expectancy have shown that obesity in adulthood is associated with a decrease in life expectancy of approximately 6–13 y [5,6]. Two United States studies using data from National Health Surveys showed that obesity in adulthood was associated not only with reduced life expectancy but also with a reduced number of years lived free of diabetes and cardiovascular disease in men and women [7,8]. Specifically, the study by Grover et al. showed that obesity in individuals aged 40–59 y was associated with a shorter life expectancy free of diabetes and cardiovascular disease by 5.9 y in men and 10.3 y in women [7]. Notably, this study did not distinguish between life expectancy with and without diabetes. The study performed by Narayan et al., which primarily focused on the effect of obesity on lifetime risk of diabetes, reported that individuals with obesity had an earlier onset of diabetes during their lifespan and spent more years lived with diabetes [8]. Nevertheless, both studies do not provide a direct observation of a well-defined population, as the results are obtained by modelling and simulation.

Therefore, we aimed to calculate the association of overweight and obesity with total life expectancy and years lived with and without diabetes at 55 y of age. We constructed multistate life tables using data collected from 1997–2001 and with over 14 y of follow-up from the Rotterdam Study.

## Methods

### Ethical Considerations

The Rotterdam Study has been approved by the medical ethics committee according to the Population Screening Act: Rotterdam Study, executed by the Ministry of Health, Welfare and Sports of the Netherlands. All participants in the present analysis provided written informed consent to participate and to obtain information from their treating physicians.

### Study Population

This study was embedded within the framework of the Rotterdam Study (RS), a prospective cohort study of the community-dwelling population in Rotterdam, Netherlands. The objectives and design of the Rotterdam Study have been described in detail elsewhere [9]. In response to demographic changes leading the acceleration of population aging, the Rotterdam Study was originally designed to investigate determinants of disease occurrence and progression in the elderly. In addition to contributing to the understanding of the etiology of geriatric illnesses, the study is expected to lead to specific recommendations for intervention. Following the pilot in 1989, recruitment started in January 1990 of all residents aged 55 y or older, of whom 7,983 (78%) agreed to participate (RS Cohort I. The study was extended in 2000, with a second cohort of individuals (RS-II) who had reached the age of 55 y or moved into the study area.

For the current study, we used data from the participants attending the third examination of the original cohort (RS-I visit 3, 1997–1999; *n* = 4,797) and the participants attending the first examination of the extended cohort (RS-II visit 1, 2000–2001; *n* = 3,011).

We excluded participants who did not visit the research center, did not have information on body mass index (BMI; *n* = 1,051) or no information on smoking behavior (*n* = 40). To account for disease-related weight loss, we excluded participants who had BMI < 18.5 (*n* = 51). Individuals without informed consent (*n* = 30) or those who did not have diabetes follow-up information (*n* = 137) were further excluded. Finally, 6,499 participants (3,656 women) were available for the current analysis.

### Assessment of Anthropometric Measurements, Health Behaviors, and Laboratory Measurements

Anthropometrics were measured in the research center by trained staff. Height and weight were measured with the participants standing without shoes and heavy outer garments. BMI was calculated as weight divided by height squared (kg/m^2^) [10]. According to the WHO cut-off criteria, we composed BMI as a categorical variable with three categories: normal weight (18.5 ≤ BMI < 25), overweight (25 ≤ BMI < 30) and obese (30 ≤ BMI).[10]. For our data analysis, obesity was grouped into a single category of BMI of 30.0 and higher because of the small sample size in each obesity class (e.g., 30 < BMI ≤ 35 and 35 < BMI < 40 and BMI ≥ 40). Smoking status was categorized as current smoker, former smoker, and never smoker, and additionally, for current smokers, we accounted for cigarettes smoked per day. Information on education was assessed according to the standard international classification of education and was composed into four categories: elementary education, lower secondary education, higher secondary education, and tertiary education [11]. Marital status was divided into single, married, widowed, or divorced/separated. Physical activity was measured by questionnaire and expressed in metabolic equivalent hours (METh)/week. For analysis, we divided the population into three equal groups (tertile) [12]. Alcohol consumption was categorized as less than 1 glass/d, 1–4 glasses/d for men and 1–2 glasses/d for women, and >4 glasses/d for men and >2 glasses/d for women. Comorbidity was considered present when “non-obesity-related cancers other than skin cancer” or chronic obstructive pulmonary disease was prevalent at baseline. From baseline comorbidities, we excluded cancers associated with obesity [13] and cancers that are curable and not likely to be related to weight loss or mortality, such as skin cancer [14]. Cancers induced by obesity are in the pathway between obesity and mortality; therefore, we accounted them as mediators. Chronic obstructive pulmonary disease was defined as a type of obstructive lung disease characterized by airflow limitation that is not fully reversible [15]. Chronic obstructive pulmonary disease has been shown to be accompanied with weight loss [16].

Hypertension, dyslipidemia, and cardiovascular disease were also considered as mediators, and therefore, we did not adjust for them in the main analyses. However, to investigate the independent association of obesity on diabetes and mortality, we conducted an additional sensitivity analysis by adjusting in the multivariable analysis for comorbidities including chronic obstructive pulmonary disease, all cancers, and cardiovascular disease at baseline. The presence of hypertension and dyslipidemia was based on medication information, whereas cardiovascular disease was defined as the presence of one or more definite manifestations of coronary heart disease (coronary revascularization, nonfatal or fatal myocardial infarction, or death due to coronary heart disease), stroke, and heart failure [17–19].

### Assessment of Outcome

Participants were followed up from the date of baseline center visit onwards. At baseline and during follow-up, cases of diabetes were ascertained by use of general practitioners’ records (including laboratory glucose measurements), hospital discharge letters, and serum glucose measurements from Rotterdam Study visits, which take place roughly every 4 y [20]. Diabetes was defined according to recent WHO guidelines [21] as a fasting serum blood glucose ≥ 7.0 mmol/L, a nonfasting blood glucose ≥ 11.1 mmol/L (when fasting samples were not available), or the use of blood-glucose-lowering medication. Information regarding the use of blood-glucose-lowering medication was ascertained from both structured home interviews and linkage to pharmacy records [21]. All potential prevalent cases of diabetes were independently reviewed by two study physicians. In case of disagreement, consensus was reached with an endocrinologist.

### Statistical Analysis

We did not publish or preregister a plan for this study. The analysis plan is described below, with any differences noted in S1 Text. To calculate the life expectancy with and without diabetes in normal weight, overweight, and obese groups, we created a multistate life table, which is a demographic tool that allows the experience of individuals in different health states to be combined in order to calculate the total life expectancy and the amount of years that individuals could expect to live in the different health states [22]. We constructed three different health states: free of diabetes, diabetes, and death. The possible transition directions were from free of diabetes to diabetes (incident diabetes), free of diabetes to death (mortality among nondiabetics), and from diabetes to death (mortality among diabetics). No backflows were allowed, and only the first event into a state was considered.

To obtain transition rates, we calculated the overall age- and sex-specific rates for each transition. Next, we calculated the prevalence of normal weight, overweight, and obesity by sex, by 10-y age groups, and separately for subjects with and without diabetes. Subsequently, we computed gender-specific hazard ratios (HRs) comparing overweight and obese individuals to normal weight individuals by using Poisson regression with “Gompertz” distribution in two models. Model 1 was adjusted for age, and Model 2 was adjusted for age, smoking status, cigarettes smoked per day (for current smokers), alcohol consumption, education, marital status, physical activity, and comorbidities (non-obesity-related cancers other than skin cancer or chronic obstructive pulmonary disease).

Finally, transition rates were calculated for each category of BMI separately using (a) the overall transition rates, (b) the adjusted HRs (model 2) for diabetes and mortality, and (c) the prevalence of normal weight, overweight, and obesity by sex and with and without diabetes. Similar calculations have been described previously [23,24]. The multistate life table started at age 55 y and closed at age 100 y.

We used Monte Carlo simulation (parametric bootstrapping) with 10,000 runs to calculate the confidence intervals of our life expectancy estimates with @RISK software (Palisade) [25].

To exclude any potential bias caused by smoking or comorbidities at baseline, we repeated the analysis among those who were both nonsmokers and without comorbidities (*n* = 5,018). Additionally, we estimated the life expectancy among participants without hypertension, dyslipidemia, and cardiovascular disease at baseline (*n* = 3,843). To account for possible reverse causation, we estimated the HRs after excluding diabetes events (*n* = 64) or deaths (*n* = 186) during the first 2 y of follow-up. Moreover, as a sensitivity analysis, we excluded the individuals with BMI < 22 (*n* = 448) to provide more conservative estimates of overweight and obesity in association with mortality.

To deal with missing values (less than 5%) for covariables including education, living situation, income, and alcohol, we used single imputation with the expectation maximization method in SPSS (IBM SPSS Statistical for Windows, Armonk, New York: IBM). This method allowed us to impute the missing values as a function of other variables by using regression methods. We used STATA version 13 for Windows (StataCorp, College Station, Texas) and R statistical software (A language and environment for statistical computing; R Foundation for Statistical Computing, Vienna, Austria) for our analysis.

## Results

The final study population consisted of 6,499 individuals: 2,843 men and 3,656 women. In total, we observed 697 (12.4%) incident diabetes events and 2,192 (33.7%) overall deaths over 14 y of follow-up. The mean age of the population was 69.2. Compared to women, men at baseline were younger, consumed higher alcohol amounts, and smoked more but showed lower levels of BMI and physical activity. While more women were on treatment for hypertension, more men were treated for dyslipidemia. Furthermore, the prevalence of cardiovascular disease and other comorbidities was higher among men (Table 1).

**Table 1:** Baseline characteristics of study population (*n* = 6,499).

Characteristics	Men	Women
Population		
*n*	2,843 (43.7)	3,656 (56.3)
Age at interview (years)	68.7 (7.9)	69.6 (8.4)
Anthropometry		
BMI, kg/m^2^	26.6 (3.2)	27.4 (4.4)
Normal (BMI 18.5–25)	927 (32.6%)	1,174 (32.1%)
Overweight (BMI 25–30)	1,525 (53.6%)	1,575 (43.1%)
Obese (BMI 30+)	391 (13.8%)	907 (24.8%)
Social Economic Status		
Marital status		
Single	83 (2.9%)	254 (6.8%)
Married	2,247 (79.0%)	1,958 (53.6%)
Widowed	306 (10.8%)	1,069 (29.2%)
Divorced/separated	207 (7.3%)	375 (10.3%)
Education		
Elementary	268 (9.4%)	599 (16.4%)
Lower secondary	853 (30.0%)	1,953 (53.4%)
Higher secondary	1,109 (39.0%)	863 (23.6%)
Tertiary	613 (21.6%)	241 (6.6%)
Lifestyle Variables		
Smoking		
Never smoker	910 (32.0%)	2,266 (62.0%)
Former smoker	1,417 (49.8%)	780 (21.3%)
Current smoker	516 (18.1%)	610 (16.7)
Daily cigarettes smoked	2.8 (7.0)	2.3 (6.1)
Alcohol (drinks/d)		
<1 glass/d	1,270 (44.7%)	2,601 (71.1%)
1–4 glasses/d (men); 1–2 glasses/d(women)	1,340 (47.1%)	658 (18.0%)
>4 glasses/d (men); >2 glasses/d(women)	233 (8.2%)	397 (10.9%)
Physical activity (METh)	74.0 (43.8)	92.6 (43.1)
Treatment for Hypertension	604 (22.2%)	909 (26.1%)
Treatment for Dyslipidemia	410 (14.4%)	456 (12.5%)
Comorbidities (Cancer ^a^ and Chronic Obstructive Pulmonary Disease)	270 (9.5%)	204 (5.6%)
Prevalence of Cardiovascular Disease	573 (20.2)	303 (8.3%)

BMI, body mass index; METh, metabolic equivalent hour. Values are means (standard deviations [SDs]) or numbers (percentages) or median (interquartile range [IQR]).

^a^ Cancer includes “non-obesity-related cancers other than skin cancer.”

### Diabetes Events and Death

Table 2 shows the HRs of the association between BMI categories with risk of incident diabetes and mortality among men and women. In multivariable adjusted model, obesity (BMI higher than 30) was associated with an increased risk of incident diabetes in men (HR 2.13, 95% CI 1.48–3.07, *p* < 0.001) and women (HR 3.54, 95% CI 2.64–4.75, *p* < 0.001) comparing with normal weight individuals (Table 2).

**Table 2:** Hazard ratios for incidence diabetes and death in overweight and obese men and women.

		Men			Women		
Transition	Categories	Cases, Number/Person-Years	Model 1 HR (95% CI)^a^	Model 2 HR (95% CI)^b^	Cases, Number/Person-Years	Model 1 HR (95% CI)^a^	Model 2 HR (95% CI)^b^
Incident T2D	Normal weight	297/23,110	1.0 (Reference)	1.0 (Reference)	400/33,152	1.0 (Reference)	1.0 (Reference)
	Overweight		1.45 (1.10–1.90)	1.52 (1.15–2.00)		2.27 (1.72–3.00)	2.32 (1.76–3.06)
	Obese		2.00 (1.40–2.87)	2.13 (1.48–3.07)		3.47 (2.60–4.65)	3.54 (2.64–4.75)
Mortality among Those without T2D	Normal weight	858/24,527	1.0 (Reference)	1.0 (Reference)	837/35,227	1.0 (Reference)	1.0 (Reference)
	Overweight		0.97 (0.84–1.13)	1.02 (0.88–1.18)		0.82 (0.71–0.96)	0.85 (0.76–0.99)
	Obese		0.96 (0.75–1.22)	1.00 (0.78–1.28)		0.86 (0.72–1.03)	0.89 (0.74–1.06)
Mortality among Those with T2D	Normal weight	335/5,259	1.0 (Reference)	1.0 (Reference)	253/6,237	1.0 (Reference)	1.0 (Reference)
	Overweight		0.90 (0.70–1.15)	0.99 (0.77–1.28)		0.75 (0.54–1.04)	0.77 (0.55–1.09)
	Obese		0.77 (0.55–1.07)	0.79 (0.56–1.11)		0.72 (0.51–1.02)	0.70 (0.55–1.01)

HR, hazard ratio; T2D, type two diabetes.

^a^ Adjusted for age.

^b^ Adjusted for age, smoking, cigarettes smoked per day for current smokers, education level, marital status, physical activity, alcohol use, and comorbidities (“non-obesity-related cancers other than skin cancer” or chronic obstructive pulmonary disease).

The association between obesity and mortality among those without diabetes was not statistically significant for both men (HR 1.00, 95% CI 0.78–1.28, *p* = 0.994) and women (HR 0.89, 95% CI 0.74–1.06, *p* = 0.198). Similarly, we did not find significant associations between obesity and mortality among individuals with diabetes. The corresponding HRs and 95% CI are 0.79 (0.56–1.11, *p* = 0.173) for men and 0.70 (0.55–1.01, *p* = 0.051) for women (Table 2).

### Total Life Expectancy and Life Expectancy with and without Diabetes

**Fig 1:**
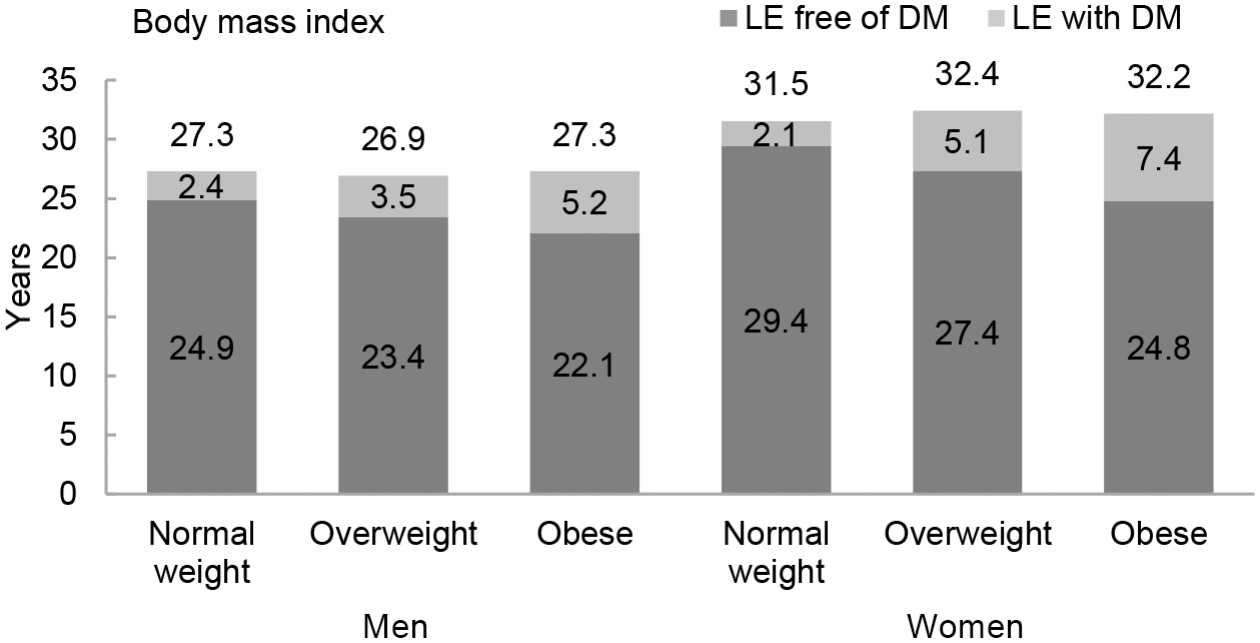
Life expectancy with and without diabetes at age 55 y for different weight categories. BMI categories: normal weight BMI is <25 kg/m^2^, overweight BMI is 25–30 kg/m2, and obese BMI is ≥30 kg/m^2^. DM, type 2 diabetes mellitus; LE, life expectancy.

The association between normal weight, overweight, and obesity with the risk of each transition was translated into number of years lived with and without diabetes (Fig 1 and Table 3). Total life expectancy for men and women with overweight and obesity were not significantly different than normal weight counterparts. Compared to normal weight men, the life expectancy of 55-y-old men in the obese group was 0.0 y (95% CI −1.3 to 1.3). For women, these differences were 0.7 (95% CI −0.3 to 1.6) y (Table 3). For both men and women, obesity was associated with fewer years lived without diabetes and more years lived with diabetes than their normal weight counterparts. Men and women with obesity lived 2.8 (95% CI −6.1 to −0.1) and 4.7 (95% CI −9.0 to −0.6) fewer y without diabetes, respectively, than those in the normal weight group. Additionally, men and women with obesity lived more years with diabetes than their normal weight counterparts: 2.8 (95% CI 0.6 to 6.2) y for men and 5.3 (95% CI 1.6 to 9.3) y for women (Fig 1 and Table 3).

**Table 3:** Differences in life expectancy, in years, at age 55 y for normal weight, overweight, and obesity in men and women.

	Total LE	Difference in Total Life Expectancy	Life Expectancy Free of Diabetes	Differences in Number of Years Lived Free of Diabetes	Life Expectancy with Diabetes	Differences in Number of Years Lived with Diabetes
Men						
Normal Weight	27.3 (26.7 to 27.9)	Ref	24.9 (24.1 to 25.7)	Ref	2.4 (1.9 to 3.0)	Ref
Overweight	26.9 (26.5 to 27.5)	−0.4 (−1.2 to 0.5)	23.4 (22.6 to 24.4)	−1.5 (−2.7 to −0.1)	3.5 (2.8 to 4.1)	1.1 (0.2 to 2.2)
Obese	27.3 (26.0 to 28.6)	0.0 (−1.3 to 1.3)	22.1 (19.1 to 24.7)	−2.8 (−6.1 to −0.1)	5.2 (3.1 to 7.9)	2.8 (0.6 to 6.2)
Women						
Normal Weight	31.5 (31.1 to32.1)	Ref	29.4 (28.4 to 30.5)	Ref	2.1 (1.3 to 2.9)	Ref
Overweight	32.4 (31.8 to 33.1)	0.9 (0.1 to 1.7)	27.4 (25.5 to 29.6)	−2.1 (−4.3 to 0.1)	5.1 (3.1 to 6.7)	3.0 (1.1 to 4.8)
Obese	32.2 (31.3 to 33.0)	0.7 (−0.3 to 1.6)	24.8 (21.1 to 28.5)	−4.7 (−9.0 to −0.6)	7.4 (4.0 to 10.8)	5.3 (1.6 to 9.3)

LE, life expectancy; Ref, Reference. We calculated the differences for total life expectancy and years lived with and without diabetes by subtracting the estimates of overweight and obese individuals from those of normal weight individuals.

Total life expectancy and number of years lived with and without diabetes for normal weight, overweight, and obese individuals who are non-smokers and without prevalent comorbidities (“non-obesity-related cancers other than skin cancer” and chronic obstructive pulmonary disease) are presented in S1 Fig, and for individuals without hypertension, dyslipidemia, and cardiovascular disease, they are presented in S2 Fig. As expected, compared to the overall population included in the main analyses, total life expectancy was higher for individuals who were nonsmokers and without comorbidities at baseline and for individuals without cardiovascular disease, hypertension, and dyslipidemia. However, differences in years lived with and without diabetes among normal weight, overweight, and obese individuals were overall similar to those found in the total population. S1 Table shows the baseline characteristics of individuals who did not visit the research center or without BMI information. Individuals in this subgroup were older than the individuals included in the study and were less physically active. Additionally, when we repeated the main analysis after excluding incident diabetes and deaths during the first 2 y of follow-up (S2 Table), excluding individuals with BMI < 22 (S3 Table), or adjusting for all comorbidities (all cancers, cardiovascular disease, and chronic obstructive pulmonary disease) (S4 Table), we found generally similar results to the main analyses.

## Discussion

Overweight and obesity at age 55 y and older represent not only a significant increase in the risk of developing diabetes but also an important decrease in the number of years lived free of diabetes and an extended number of years lived with diabetes when compared with normal weight counterparts. While total life expectancy remained unaffected, on average, obesity was associated with 2.8 fewer y lived free from diabetes in men and 4.7 fewer y in women. Additionally, obese men and women respectively lived 2.8 and 5.3 y longer with diabetes compared to their normal weight counterparts.

Years lived free of diabetes are a result of two components: incidence of diabetes and mortality in those without diabetes. We observed a higher risk of incident diabetes in overweight and obese individuals when compared to their normal weight counterparts, which could reflect an earlier diagnosis of diabetes across lifespan. Furthermore, a higher risk of mortality in those without diabetes will result in a decrease of total life expectancy and consequently will shorten years lived free of diabetes. The number of years lived with diabetes is a consequence of incident diabetes risk and mortality risk among those with diabetes. Higher incidence of diabetes would lead to an earlier occurrence of diabetes, whereas lower risk of mortality among those with diabetes would lead to greater number of years lived with diabetes.

Our analysis indicated that overweight and obesity increased the risk of diabetes for both men and women, and the HRs were comparable with other studies [26,27]. Additionally, we showed that overweight and obesity were not associated with mortality in individuals with and without diabetes. A recent meta-analysis including diabetic populations revealed a lower risk of mortality among overweight and obese subjects than normal weight counterparts [28]. Although our estimates of mortality risk among diabetic patients are similar to the meta-analysis, we cannot support the protective effect of obesity on mortality until further research is done.

In our study, total life expectancy in individuals aged 55 y and over for both men and women remained unaffected by overweight and obesity. In contrast, an earlier study using Framingham Study data has showed that at the age of 40 y, obesity was associated with large decreases in total life expectancy [6]. This discrepancy could be explained by the difference in participants’ age (55 versus 40) in life expectancy calculations and differences in the calendar time of baseline measurements (1997 versus 1948). Given the improvements in prevention and treatment of cardiometabolic risk factors in the last decade, the association of obesity with mortality has diminished substantially [29,30]. Consistent with our findings, recent data among the middle-aged and elderly has demonstrated that overweight and obesity are not associated with a reduction in life expectancy [31,32]. Nevertheless, our study extended the previous evidence by calculating the association of obesity in life expectancy with and without diabetes. We demonstrated that obesity increases the risk of developing diabetes earlier in life and further extends years lived with diabetes. These findings support previous results from a study by Narayan et al. [8], which used data from the US National Health Survey. However, our study is unique regarding the approach used for estimating life expectancy with and without diabetes. While Narayan et al. obtained the estimates by modelling and simulation, we calculated the life expectancy with and without diabetes from direct observation of a well-defined population using multistate life tables. Moreover, the study by Narayan et al. used self-reported data for the diagnosis of diabetes and information on height and weight, whereas in our study we had well-ascertained diabetes diagnoses obtained from physicians and linkage to pharmacy data and weight and height measured by trained research assistants at the study center.

In our study, we noted a difference in the number of years lived with diabetes among men and women. Compared to men, women had an increased risk of diabetes by BMI, indicating an earlier occurrence of diabetes during their life. Moreover, women with diabetes had a lower risk of mortality compared to men. Taken these results together, we could explain why women spend more years with diabetes than men. This is in accordance with previous research conducted in the US concluding that women spend more years living with diabetes than men [8], possibly due to larger differences in probabilities of death between males and females observed for patients with diabetes relative to those without diabetes [33].

Strengths of the current study include the use of data from a prospective, well-organized study with long-term follow-up. The diagnosis of incident diabetes was done by standardized blood glucose measurements at the repeated study center visits and electronic linkage with pharmacy dispensing records in the study area. Height and weight were measured in the research center by trained staff. Nevertheless, some limitations of this study should be addressed. In our analysis, we excluded individuals with missing information on weight and height since the BMI is our main exposure. This subgroup was older and less physically active. Furthermore, since the generalizability of these findings could be limited to middle-aged and older white European populations, our results need confirmation in other populations. Additionally, studies evaluating the association of obesity with mortality could be prone to incorrect adjustment for confounders such as smoking or weight loss related to comorbidities. In our study, we adjusted for smoking status and the cigarettes smoked per day and comorbidities. Moreover, we conducted a sensitivity analysis to take into account reverse causation by excluding events during the first 2 y of follow-up.

The added value of this study is the combination of the observed effects of obesity on diabetes incidence and mortality translated into population measures such as life expectancy with and without diabetes that might be important to clinicians, patients, and policy makers in tackling the next stages of obesity epidemics. Our study showed that among middle-aged and elderly individuals, total life expectancy was not different for those who were overweight or obese. Obesity is associated with earlier and extended periods lived with diabetes. Those extra years of life will be filled with an expansion of accompanying comorbidities, placing a higher toll on clinicians and health care systems and challenging the new global strategies for obesity and diabetes prevention.

## Supporting Information

S1 Fig Life expectancy with and without diabetes at age 55 y in nonsmokers and without comorbidities for different weight categories^a^. BMI categories: normal weight BMI is <25 kg/m^2^, overweight BMI is 25–30 kg/m^2^, and obese BMI is ≥30 kg/m^2^. DM, type 2 diabetes mellitus; LE, life expectancy. ^a^ Comorbidity was considered present when “non-obesity-related cancers other than skin cancer” or chronic obstructive pulmonary disease was prevalent at baseline.
(TIF)

S2 Fig Life expectancy with and without diabetes at age 55 y in subject without hypertension, dyslipidemia, and cardiovascular disease for different weight categories. BMI categories: normal weight BMI is <25 kg/m^2^, overweight BMI is 25–30 kg/m^2^, and obese BMI is ≥30 kg/m^2^. DM, diabetes mellitus; LE, life expectancy.
(TIF)

S1 STROBE Checklist Strengthening the Reporting of Observational Studies in Epidemiology (STROBE) Checklist. (DOCX)

S1 Table Baseline characteristics^a^ of individuals who did not visit the research center or did not have information on BMI (*n* = 1,051). Values are means (standard deviations [SDs]) or numbers (percentages). ^a^ Baseline characteristics are based in home interview. ^b^ Cancer includes “non-obesity-related cancers other than skin cancer.”
(DOCX)

S2 Table HRs for diabetes and death for overweight and obese men and women, excluding the first 2 y of follow up for death and incidence diabetes. ^a^ Adjusted for age. ^b^ Adjusted for age, smoking, cigarettes smoked per day, education level, marital status, physical activity, alcohol use, and comorbidities (“non-obesity-related cancers other than skin cancer” or chronic obstructive pulmonary disease).
(DOCX)

S3 Table HRs for diabetes and death for overweight and obese men and women in subjects with BMI > 22. ^a^ Adjusted for age. ^b^Adjusted for age, smoking, cigarettes smoked per day, education level, marital status, physical activity, alcohol use, and comorbidities (“non-obesity-related cancers other than skin cancer” or chronic obstructive pulmonary disease).
(DOCX)

S4 Table HRs for diabetes and death for overweight and obese men and women adjusting for all comorbidities. ^a^ Adjusted for age, smoking, cigarettes smoked per day for current smokers, education level, marital status, physical activity, alcohol use, and comorbidities (all cancers, cardiovascular disease, and chronic obstructive pulmonary disease).
(DOCX)

S1 Text Outline of changes made to the analysis plan. (DOCX)

## Abbreviations

BMI: body mass index
HR: hazard ratio
IQR: interquartile range
METh: metabolic equivalent hour
RS: Rotterdam Study
SD: standard deviation
STROBE: Strengthening the Reporting of Observational Studies in Epidemiology
T2D: type two diabetes

## References

[1] Collaboration NCDRF, Di Cesare M, Bentham J, Stevens GA, Zhou B, Danaei G, et al Trends in adult body-mass index in 200 countries from 1975 to 2014: a pooled analysis of 1698 population-based measurement studies with 19.2 million participants. Lancet. 2016;387(10026):1377–96. Epub 2016/04/27. S0140-6736(16)30054-X [pii] 10.1016/S0140-6736(16)30054-X . 27115820 PMC7615134

[2] Mokdad AH, Ford ES, Bowman BA, Dietz WH, Vinicor F, Bales VS, et al Prevalence of obesity, diabetes, and obesity-related health risk factors, 2001. JAMA. 2003;289(1):76–9. Epub 2002/12/31. jbr20304 [pii]. . 12503980 10.1001/jama.289.1.76

[3] Epstein RM, Alper BS, Quill TE. Communicating evidence for participatory decision making. Jama-J Am Med Assoc. 2004;291(19):2359–66. 10.1001/jama.291.19.2359 ISI:000221455400024. 15150208

[4] Leal J, Gray AM, Clarke PM. Development of life-expectancy tables for people with type 2 diabetes. Eur Heart J. 2009;30(7):834–9. 10.1093/eurheartj/ehn567 ISI:000264889600019. 19109355 PMC2663724

[5] Fontaine KR, Redden DT, Wang CX, Westfall AO, Allison DB. Years of life lost due to obesity. Jama-J Am Med Assoc. 2003;289(2):187–93. 10.1001/jama.289.2.187 ISI:000180226400029. 12517229

[6] Peeters A, Barendregt JJ, Willekens F, Mackenbach JP, Al Mamun A, Bonneux L, et al Obesity in adulthood and its consequences for, life expectancy: A life-table analysis. Ann Intern Med. 2003;138(1):24–32. ISI:000180996200004. 12513041 10.7326/0003-4819-138-1-200301070-00008

[7] Grover SA, Kaouache M, Rempel P, Joseph L, Dawes M, Lau DCW, et al Years of life lost and healthy life-years lost from diabetes and cardiovascular disease in overweight and obese people: a modelling study. Lancet Diabetes Endo. 2015;3(2):114–22. 10.1016/S2213-8587(14)70229-3 ISI:000353030900018. 25483220

[8] Narayan KM, Boyle JP, Thompson TJ, Gregg EW, Williamson DF. Effect of BMI on lifetime risk for diabetes in the U.S. Diabetes Care. 2007;30(6):1562–6. Epub 2007/03/21. dc06-2544 [pii] 10.2337/dc06-2544 . 17372155

[9] Hofman A, Brusselle GGO, Murad SD, van Duijn CM, Franco OH, Goedegebure A, et al The Rotterdam Study: 2016 objectives and design update. Eur J Epidemiol. 2015;30(8):661–708. 10.1007/s10654-015-0082-x ISI:000361751700007. 26386597 PMC4579264

[10] Eveleth PB. Physical status: The use and interpretation of anthropometry. Report of a WHO Expert Committee—WHO. Am J Hum Biol. 1996;8(6):786–7. 10.1002/(Sici)1520-6300(1996)8:6<786::Aid-Ajhb11>3.0.Co;2-I ISI:A1996VZ64700011.

[11] Unesco. International Standard Classification of Education. Unesco, November 1997.

[12] Koolhaas CM, Dhana K, Golubic R, Schoufour JD, Hofman A, van Rooij FJ, et al Physical Activity Types and Coronary Heart Disease Risk in Middle-Aged and Elderly Persons: The Rotterdam Study. Am J Epidemiol. 2016 Epub 2016/03/30. kwv244 [pii] 10.1093/aje/kwv244 . 27022033

[13] Wiseman M. The second World Cancer Research Fund/American Institute for Cancer Research expert report. Food, nutrition, physical activity, and the prevention of cancer: a global perspective. Proc Nutr Soc. 2008;67(3):253–6. Epub 2008/05/03. S002966510800712X [pii] 10.1017/S002966510800712X . 18452640

[14] Leiter U, Eigentler T, Garbe C. Epidemiology of skin cancer. Adv Exp Med Biol. 2014;810:120–40. Epub 2014/09/11. . 25207363 10.1007/978-1-4939-0437-2_7

[15] van Durme YM, Verhamme KM, Stijnen T, van Rooij FJ, Van Pottelberge GR, Hofman A, et al Prevalence, incidence, and lifetime risk for the development of COPD in the elderly: the Rotterdam study. Chest. 2009;135(2):368–77. Epub 2009/02/10. S0012-3692(09)60124-0 [pii] 10.1378/chest.08-0684 . 19201711

[16] Agusti AG, Sauleda J, Miralles C, Gomez C, Togores B, Sala E, et al Skeletal muscle apoptosis and weight loss in chronic obstructive pulmonary disease. Am J Respir Crit Care Med. 2002;166(4):485–9. Epub 2002/08/21. 10.1164/rccm.2108013 . 12186825

[17] Kavousi M, Elias-Smale S, Rutten JH, Leening MJ, Vliegenthart R, Verwoert GC, et al Evaluation of newer risk markers for coronary heart disease risk classification: a cohort study. Ann Intern Med. 2012;156(6):438–44. Epub 2012/03/21. 156/6/438 [pii] 10.7326/0003-4819-156-6-201203200-00006 . 22431676

[18] Bos MJ, Koudstaal PJ, Hofman A, Ikram MA. Modifiable etiological factors and the burden of stroke from the Rotterdam study: a population-based cohort study. PLoS Med. 2014;11(4):e1001634 Epub 2014/05/02. 10.1371/journal.pmed.1001634PMEDICINE-D-13-03607 [pii]. 24781247 PMC4004543

[19] Alberts VP B M, Koudstaal PJ, Hofman A, Witteman JCM, Stricker BHC. Heart failure and the risk of stroke: the Rotterdam Study. Eur Heart J. 2010;25(11):807–12. 10.1007/s10654-010-9520-y PMC2991556 21061046

[20] Leening MJG, Kavousi M, Heeringa J, van Rooij FJA, Verkroost-van Heemst J, Deckers JW, et al Methods of data collection and definitions of cardiac outcomes in the Rotterdam Study. Eur J Epidemiol. 2012;27(3):173–85. 10.1007/s10654-012-9668-8 ISI:000305218800003. 22388767 PMC3319884

[21] Organization. WH. Definition and diagnosis of diabetes mellitus and intermediate hyperglycemia: Report of a WHO/IDF consultation. Geneva: World Health Organization, 2006.

[22] Schoen R. Modeling multigroup populations: Springer Science & Business Media; 2013.

[23] Franco OH, de Laet C, Peeters A, Jonker J, Mackenbach J, Nusselder W. Effects of physical activity on life expectancy with cardiovascular disease. Arch Intern Med. 2005;165(20):2355–60. 10.1001/archinte.165.20.2355 ISI:000233251900006. 16287764

[24] Franco OH, Steyerberg EW, Hu FB, Mackenbach J, Nusselder W. Associations of diabetes mellitus with total life expectancy and life expectancy with and without cardiovascular disease. Arch Intern Med. 2007;167(11):1145–51. 10.1001/archinte.167.11.1145 ISI:000247143400006. 17563022

[25] Bradley Efron RJT. An Introduction to the Bootstrap. New York, NY: Chapman & Hall; 1993.

[26] Tuomilehto J, Lindstrom J, Eriksson JG, Valle TT, Hamalainen H, Ilanne-Parikka P, et al Prevention of type 2 diabetes mellitus by changes in lifestyle among subjects with impaired glucose tolerance. New Engl J Med. 2001;344(18):1343–50. 10.1056/Nejm200105033441801 ISI:000168413500001. 11333990

[27] Knowler WC, Barrett-Connor E, Fowler SE, Hamman RF, Lachin JM, Walker EA, et al Reduction in the incidence of type 2 diabetes with lifestyle intervention or metformin. New Engl J Med. 2002;346(6):393–403. ISI:000173686400002. 11832527 10.1056/NEJMoa012512 PMC1370926

[28] Chang HW L Y, Hsieh CH, Liu PY, Lin GM. Association of body mass index with all-cause mortality in patients with diabetes: a systemic review and meta-analysis. Cardiovasc Diagn Ther 2016;6(2):109–19. 10.21037/cdt.2015.12.06 27054100 PMC4805755

[29] Gregg EW, Cheng YJ, Cadwell BL, Imperatore G, Williams DE, Flegal KM, et al Secular trends in cardiovascular disease risk factors according to body mass index in US adults. JAMA. 2005;293(15):1868–74. Epub 2005/04/21. 293/15/1868 [pii] 10.1001/jama.293.15.1868 . 15840861

[30] Stevens J, Cai J, Pamuk ER, Williamson DF, Thun MJ, Wood JL. The effect of age on the association between body-mass index and mortality. N Engl J Med. 1998;338(1):1–7. . 9414324 10.1056/NEJM199801013380101

[31] Finkelstein EA, Brown DS, Wrage LA, Allaire BT, Hoerger TJ. Individual and aggregate years-of-life-lost associated with overweight and obesity. Obesity (Silver Spring). 2010;18(2):333–9. Epub 2009/08/15. oby2009253 [pii] 10.1038/oby.2009.253 . 19680230

[32] Reuser M B L, Willekens F.. The burden of mortality of obesity at middle and old age is small. A life table analysis of the US Health and Retirement Survey. Eur J Epidemiol. 2008;23(9):601–7. 10.1007/s10654-008-9269-8 18584293

[33] Gregg EW C Y, Saydah S, et al Trends in death rates among U.S. adults with and without diabetes between 1997 and 2006: findings from the National Health Interview Survey. Diabetes Care. 2012;35:1252–7. 10.2337/dc11-1162 22619288 PMC3357247

