# Supplementary figures and images for: Obesity and Life Expectancy with and without Diabetes in Adults Aged 55 Years and Older in the Netherlands: A Prospective Cohort Study
Source: PLoS Med. 2016 Jul 19;13(7):e1002086. doi: 10.1371/journal.pmed.1002086 (PMC4951120; doi:10.1371/journal.pmed.1002086)

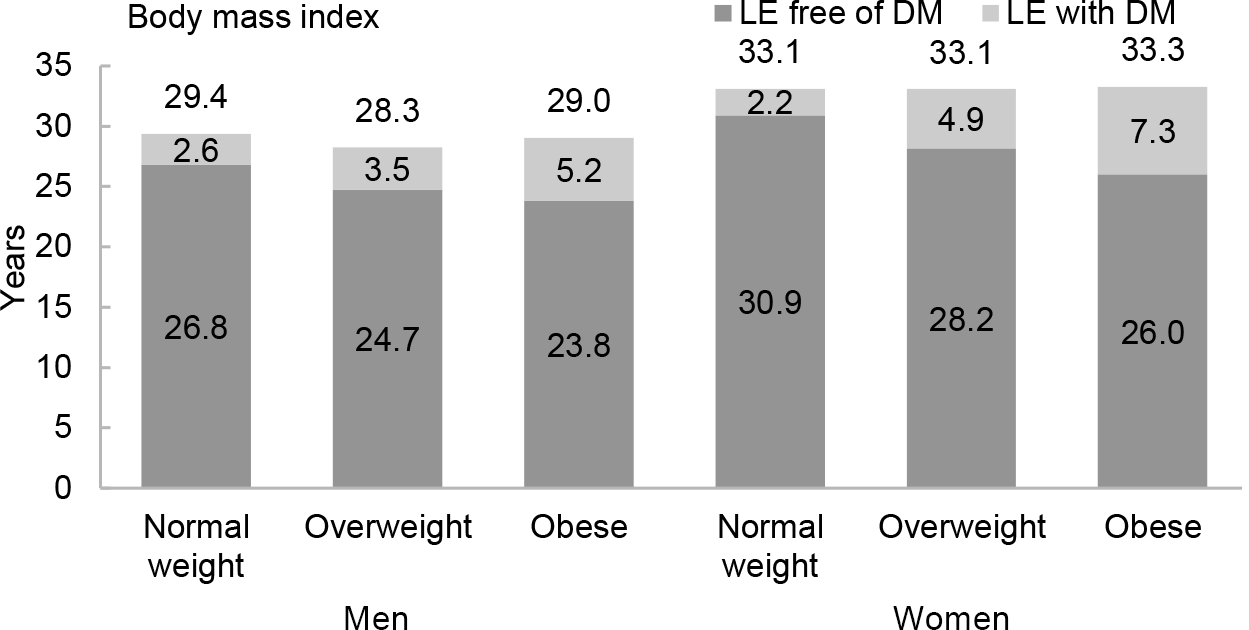

Supplement: S1 Fig — BMI categories: normal weight BMI is <25 kg/m2, overweight BMI is 25–30 kg/m2, and obese BMI is ≥30 kg/m2. DM, type 2 diabetes mellitus; LE, life expectancy. a Comorbidity was considered present when “non-obesity-related cancers other than skin cancer” or chronic obstructive pulmonary disease was prevalent at baseline. (TIF) [file pmed.1002086.s001.tif]

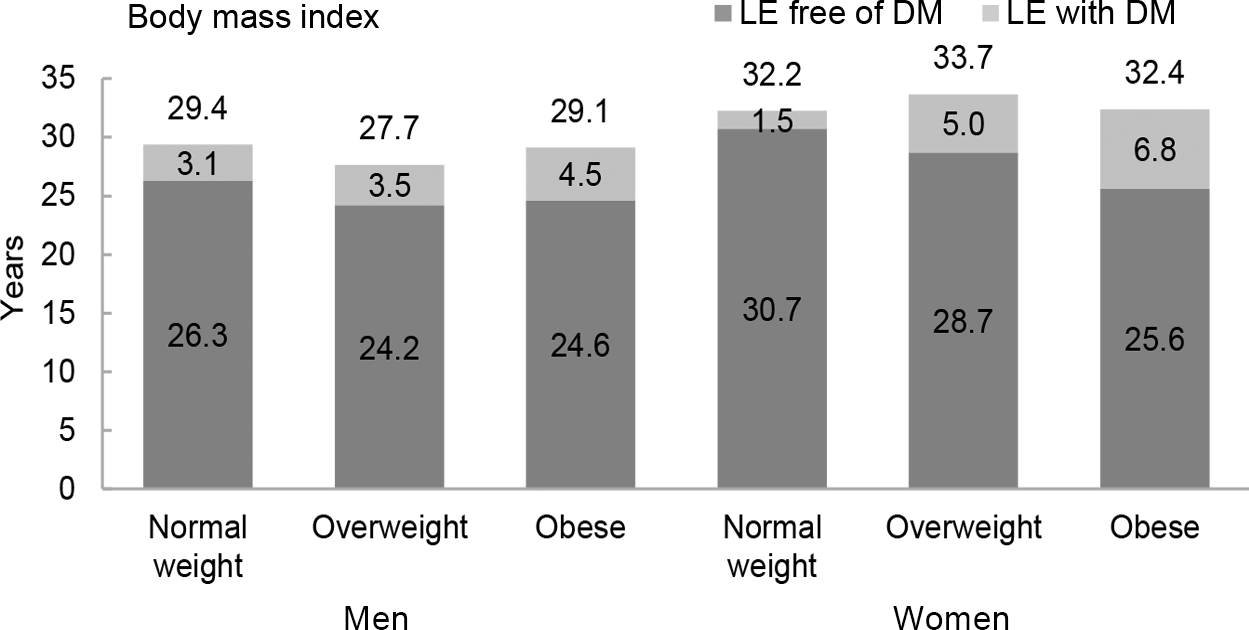

Supplement: S2 Fig — BMI categories: normal weight BMI is <25 kg/m2, overweight BMI is 25–30 kg/m2, and obese BMI is ≥30 kg/m2. DM, diabetes mellitus; LE, life expectancy. (TIF) [file pmed.1002086.s002.tif]
