# Supplementary material for: Obesity and Life Expectancy with and without Diabetes in Adults Aged 55 Years and Older in the Netherlands: A Prospective Cohort Study
Source: PLoS Med. 2016 Jul 19;13(7):e1002086. doi: 10.1371/journal.pmed.1002086 (PMC4951120; doi:10.1371/journal.pmed.1002086)
Supplement: S1 Table — Values are means (standard deviations [SDs]) or numbers (percentages). a Baseline characteristics are based in home interview. b Cancer includes “non-obesity-related cancers other than skin cancer.” (DOCX) [file pmed.1002086.s004.docx]

| S1 Table. Baseline characteristics^a^ of individuals who did not visit the research center or did not have information on BMI (n=1,051). | | |
| --- | --- | --- |
| Characteristics | Men | Women |
| Population |  |  |
| n | 312 (42%) | 739 (58%) |
| Age at interview (years) | 73.6±9.7 | 77.1±10.8 |
| Social economic status |  |  |
| *Marital status* |  |  |
| Single | 11 (3.5) | 55 ( 7.4) |
| Married | 218 (69.9) | 248 (33.6) |
| Widowed | 61 (19.5) | 372 (50.3) |
| Divorced/separated | 22 (7.1) | 64 ( 8.7) |
| *Education* |  |  |
| Elementary | 47 (15.1) | 236 (31.9) |
| Lower secondary | 99 (31.7) | 346 (46.8) |
| Higher secondary | 117 (37.5) | 115 (15.6) |
| Tertiary | 49 (15.7) | 42 ( 5.7) |
| Lifestyle variables |  |  |
| *Smoking* |  |  |
| Never smoking | 61 (21.0) | 405 (62.8) |
| Former smoker | 158 (54.3) | 122 (18.9) |
| Current smoker | 72 (24.7) | 118 (18.3) |
| Daily cigarettes smoked | 3.8±8.4 | 2.3±5.8 |
| *Alcohol* (drinks/day) |  |  |
| < 1 glass/day | 146 (46.8) | 533 (72.1) |
| 1-4 glasses/day (men); 1-2 glasses/day (women) | 141 (45.2) | 131 (17.7) |
| > 4 glasses/day (men); > 2 glasses/day (women) | 25 ( 8.0) | 75 (10.1) |
| Physical activity (METh) | 56.4±40.2 | 68.5±43.3 |
| *Treatment for hypertension* | 75 (25.3) | 195 (28.1) |
| *Treatment for dyslipidemia* | 36 (11.5) | 55 ( 7.4) |
| *Comorbidities (cancer^b^and chronic obstructive pulmonary disease)* | 48 (15.4) | 51 (6.9) |
| *CVD prevalence* | 91 (29.2) | 166 (22.5) |

Values are means (SDs) or numbers (percentages). BMI, body mass index.

^a^ Baseline characteristics are based in home interview

^b^ Cancer includes “non-obesity related cancers other than skin cancer”
