# Supplementary material for: Obesity and Life Expectancy with and without Diabetes in Adults Aged 55 Years and Older in the Netherlands: A Prospective Cohort Study
Source: PLoS Med. 2016 Jul 19;13(7):e1002086. doi: 10.1371/journal.pmed.1002086 (PMC4951120; doi:10.1371/journal.pmed.1002086)
Supplement: S2 Table — a Adjusted for age. b Adjusted for age, smoking, cigarettes smoked per day, education level, marital status, physical activity, alcohol use, and comorbidities (“non-obesity-related cancers other than skin cancer” or chronic obstructive pulmonary disease). (DOCX) [file pmed.1002086.s005.docx]

| S2 Table. Hazard ratios for diabetes and death for overweight and obese men and women, excluding the first 2 years of follow up for death and diabetes | | | | | | | |
| --- | --- | --- | --- | --- | --- | --- | --- |
|  |  | Men (n =2706) | | | Women (n= 3540) | | |
| Transition | Categories | Cases, No. / Person-Years | Model 1 HR  (95% CI)^a^ | Model 2 HR  (95% CI)^b^ | Cases, No. / Person-Years | Model 1 HR  (95% CI)^a^ | Model 2 HR  (95% CI)^b^ |
| Incident diabetes | Normal weight | 272/22967 | 1.0 Reference | 1.0 Reference | 361/33023 | 1.0 Reference | 1.0 Reference |
|  | Overweight |  | 1.49 (1.12-1.98) | 1.56 (1.17-2.08) |  | 2.28 (1.70-3.04) | 2.33 (1.75-3.13) |
|  | Obese |  | 1.93 (1.31-2.83) | 2.04 (1.38-3.02) |  | 3.36 (2.47-4.55) | 3.46 (2.54-4.71) |
| Mortality among those without diabetes | Normal weight | 756/24173 | 1.0 Reference | 1.0 Reference | 767/34737 | 1.0 Reference | 1.0 Reference |
|  | Overweight |  | 1.02 (0.88-1.19) | 1.06 (0.91-1.25) |  | 0.80 (0.68-0.94) | 0.83 (0.70-0.98) |
|  | Obese |  | 1.05 (0.81-1.35) | 1.12 (0.86-1.45) |  | 0.84 (0.70-1.02) | 0.88 (0.73-1.07) |
| Mortality among those with diabetes | Normal weight | 299/5020 | 1.0 Reference | 1.0 Reference | 232/5860 | 1.0 Reference | 1.0 Reference |
|  | Overweight |  | 0.88 (0.68-1.15) | 0.95 (0.73-1.24) |  | 0.74 (0.52-1.04) | 0.77 (0.54-1.11) |
|  | Obese |  | 0.76 (0.53-1.09) | 0.82 (0.57-1.17) |  | 0.72 (0.50-1.03) | 0.70 (0.49-1.02) |

^a^ Adjusted for age.

^b^ Adjusted for age, smoking, cigarettes smoked per day for current smokers, education level, marital status, physical activity, alcohol use and comorbidities (COPD and cancer not caused by obesity).
