# Supplementary material for: Obesity and Life Expectancy with and without Diabetes in Adults Aged 55 Years and Older in the Netherlands: A Prospective Cohort Study
Source: PLoS Med. 2016 Jul 19;13(7):e1002086. doi: 10.1371/journal.pmed.1002086 (PMC4951120; doi:10.1371/journal.pmed.1002086)
Supplement: S3 Table — a Adjusted for age. bAdjusted for age, smoking, cigarettes smoked per day, education level, marital status, physical activity, alcohol use, and comorbidities (“non-obesity-related cancers other than skin cancer” or chronic obstructive pulmonary disease). (DOCX) [file pmed.1002086.s006.docx]

| S3 Table. Hazard ratios for diabetes and death for overweight and obese men and women in subjects with BMI > 22 | | | | | | | |
| --- | --- | --- | --- | --- | --- | --- | --- |
|  |  | Men (n=2,670) | | | Women (n=3,381) | | |
| Transition | Categories | Cases, No. / Person-Years | Model 1 HR  (95% CI)^a^ | Model 2 HR  (95% CI) ^b^ | Cases, No. / Person-Years | Model 1 HR  (95% CI)^a^ | Model 2 HR  (95% CI)^b^ |
| Incident diabetes | Normal weight | 285/21,738 | 1.0 Reference | 1.0 Reference | 387/30,437 | 1.0 Reference | 1.0 Reference |
|  | Overweight |  | 1.42 (1.06-1.89) | 1.49 (1.11-2.00) |  | 2.15 (1.60-2.91) | 2.17 (1.61-2.94) |
|  | Obese |  | 1.95 (1.34-2.83) | 2.07 (1.42-3.03) |  | 3.31 (2.42-4.51) | 3.29 (2.40-4.52) |
| Mortality among those without diabetes | Normal weight | 776/23,105 | 1.0 Reference | 1.0 Reference | 755/32,428 | 1.0 Reference | 1.0 Reference |
|  | Overweight |  | 1.11 (0.95-1.30) | 1.15 (0.98-1.34) |  | 0.84 (0.71-0.99) | 0.86 (0.73-1.02) |
|  | Obese |  | 1.09 (0.85-1.40) | 1.13 (0.88-1.46) |  | 0.87 (0.72-1.03) | 0.90 (0.74-1.09) |
| Mortality among those with diabetes | Normal weight | 315/5,131 | 1.0 Reference | 1.0 Reference | 246/6,050 | 1.0 Reference | 1.0 Reference |
|  | Overweight |  | 1.01 (0.77-1.33) | 1.10 (0.83-1.45) |  | 0.64 (0.45-0.91) | 0.64 (0.44-0.91) |
|  | Obese |  | 0.87 (0.61-1.23) | 0.88 (0.61-1.24) |  | 0.62 (0.43-89) | 0.58 (0.40-0.83) |

^a^ Adjusted for age.

^b^ Adjusted for age, smoking, cigarettes smoked per day for current smokers, education level, marital status, physical activity, alcohol use and comorbidities (COPD and cancer not caused by obesity).
