# Supplementary material for: Obesity and Life Expectancy with and without Diabetes in Adults Aged 55 Years and Older in the Netherlands: A Prospective Cohort Study
Source: PLoS Med. 2016 Jul 19;13(7):e1002086. doi: 10.1371/journal.pmed.1002086 (PMC4951120; doi:10.1371/journal.pmed.1002086)
Supplement: S4 Table — a Adjusted for age, smoking, cigarettes smoked per day for current smokers, education level, marital status, physical activity, alcohol use, and comorbidities (all cancers, cardiovascular disease, and chronic obstructive pulmonary disease). (DOCX) [file pmed.1002086.s007.docx]

| S4 Table. Hazard ratios for diabetes and death for overweight and obese men and women adjusting for all comorbidities. | | | | | |
| --- | --- | --- | --- | --- | --- |
|  | Men | | | Women | |
| Transition | Categories | Cases,  No. / Person-Years | Model  HR (95% CI)^a^ | Cases,  No. / Person-Years | Model  HR (95% CI)^a^ |
| Incident diabetes | Normal weight | 297/23110 | 1.0 Reference | 400/ 33152 | 1.0 Reference |
|  | Overweight |  | 1.51 (1.15, 1.99) |  | 2.33 (1.77-3.08) |
|  | Obese |  | 2.12 (1.47, 3.06) |  | 3.55 (2.64-4.76) |
| Mortality among those without diabetes | Normal weight | 858/24527 | 1.0 Reference | 837/35227 | 1.0 Reference |
|  | Overweight |  | 0.98 (0.85-1.13) |  | 0.89 (0.76-1.04) |
|  | Obese |  | 0.99 (0.78-1.27) |  | 0.92 (0.77-1.11) |
| Mortality among those with diabetes | Normal weight | 335/5259 | 1.0 Reference | 253/6237 | 1.0 Reference |
|  | Overweight |  | 0.97 (0.75-1.25) |  | 0.78 (0.55-1.09) |
|  | Obese |  | 0.78 (0.56-1.10) |  | 0.70 (0.49-1.01) |

^a^ Adjusted for age, smoking, cigarettes smoked per day for current smokers, education level, marital status, physical activity, alcohol use and comorbidities (all cancers, cardiovascular disease and chronic obstructive pulmonary disease).
