# Supplementary material for: Obesity and Life Expectancy with and without Diabetes in Adults Aged 55 Years and Older in the Netherlands: A Prospective Cohort Study
Source: PLoS Med. 2016 Jul 19;13(7):e1002086. doi: 10.1371/journal.pmed.1002086 (PMC4951120; doi:10.1371/journal.pmed.1002086)
Supplement: S1 Text — (DOCX) [file pmed.1002086.s008.docx]

**S1 Text.** Analysis plan for the observational study described in: Dhana K, Nano J, Ligthart S, Peeters A, Hofman A, Nusselder W, Dehghan A, Franco OH. Obesity in older adults and life expectancy with and without diabetes: a prospective cohort study. PLOS Medicine.

We did not publish or pre-register a protocol for the analysis of data. As described in the methods section, we followed a clear analysis plan.

a) The study was motivated by the question “What is the effect of obesity on years lived with and without type 2 diabetes?” which addressed an important health for clinicians, patients and policy makers. As described in the manuscript, we excluded participant who did not visit the research center, did not have information on BMI, smoking behavior, diabetes follow-up or those individuals without informed consent. Furthermore, individuals who had BMI <18.5 were excluded.

b) The statistical approach was determined at the beginning of the project and did not change throughout. Multistate life tables were created to calculate the differences in life expectancy and years lived with and without diabetes in normal weight, overweight and obese groups as defined by BMI.

c) Following feedback from the reviewers, we conducted a series of sensitivity analyses. To disentangle the suggested U relationship between BMI and mortality, we excluded individuals with BMI lower than 22. To minimize the effect of reverse causation, we repeated our analysis by excluding diabetes events (first transition) or deaths (second and third transition) during the first 2 years of follow-up based on the assumption that such events are most likely due to preceding diseases.

We further considered carefully the adjustment for confounder in our models. For example, although we adjusted for comorbidities (cancers not related to obesity and chronic obstructive pulmonary disease) and smoking behavior in the main analysis, we computed lifetables by excluding participants that had comorbidities or were current smokers as a secondary analysis. Moreover, we did not adjust for hypertension, dyslipidemia or cardiovascular disease since those factors might be considered intermediate factors rather than confounders in the association between obesity and mortality. However, the analysis was repeated among participants without hypertension, dyslipidemia and cardiovascular disease at baseline. Finally, to assess the independent effect of obesity on the risk of diabetes and mortality we did an additional analysis by adjusting for all comorbidities including cardiovascular disease, all cancers and chronic obstructive pulmonary disease.
